# Technical Notes on Liver Elastography: A Guide for Use in Neonates in Intensive Care Units

**DOI:** 10.3390/jcm14051435

**Published:** 2025-02-21

**Authors:** Ángel Lancharro Zapata, Alejandra Aguado del Hoyo, María del Carmen Sánchez Gómez de Orgaz, Miguel A. Ortega, Juan Antonio León Luís

**Affiliations:** 1 Diagnostic Imaging Department, Paediatric Radiology Section, Gregorio Marañón General University Hospital, 28009 Madrid, Spain; angelmaria.lancharro@salud.madrid.org (Á.L.Z.); alejandra.aguado@salud.madrid.org (A.A.d.H.); 2 Maternal and Infant Research Unit Alonso Family Foundation (UDIMIFFA), Gregorio Marañón Health Research Institute (IiSGM), 28009 Madrid, Spain; msgomezdeorgaz@salud.madrid.org (M.d.C.S.G.d.O.); jaleon@ucm.es (J.A.L.L.); 3 Department of Neonatology, Gregorio Marañón General University Hospital, 28009 Madrid, Spain; 4 Department of Medicine and Medical Specialties, Faculty of Medicine and Health Sciences, University of Alcalá, 28801 Alcalá de Henares, Spain; 5 Ramón y Cajal Institute for Health Research (IRYCIS), 28034 Madrid, Spain; 6 Department of Public and Maternal-Child Health, Faculty of Medicine, Complutense University of Madrid, 28040 Madrid, Spain; 7 Obstetrics and Gynecology Service, Gregorio Marañón University Hospital, 28009 Madrid, Spain

**Keywords:** neonatal liver elastography, neonatal care, liver stiffness, liver pSWE, liver 2D-SWE, point-of-care ultrasound

## Abstract

**Background/Objectives:** Liver elastography is increasingly used in neonatal intensive care units (NICUs) as a non-invasive, radiation-free, reproducible technique for assessing liver stiffness. This technique demonstrates substantial advantages over conventional ultrasound in diagnosing diffuse liver diseases by providing quantitative measures of tissue elasticity. This article aims to describe the most critical milestones for performing liver elastography ultrasound point-of-care, a tool increasingly used to complement traditional ultrasound in the study of the liver in intensive care units where the population is very susceptible to manipulation. **Methods:** Techniques such as point-shear wave elastography (pSWE) and two-dimensional shear wave elastography (2D-SWE) have become key in evaluating conditions such as hypoxic-ischemic liver disease, cholestatic diseases, storage and metabolic disorders, or infectious liver conditions. However, despite its usefulness, performing elastography in neonates, particularly in those weighing less than 1000 g or in high-frequency oscillatory ventilation, presents notable challenges, including the extreme sensitivity of neonates to touch, noise, and temperature changes and the difficulty in obtaining accurate measurements due to limited hepatic depth. **Results:** Key factors for the success of sonoelastography in this population include minimizing contact time, adjusting mechanical and thermal indices to meet biosecurity guidelines, and ensuring patient comfort and stability during the procedure. Despite these challenges, elastography has proven helpful in routine clinical practice. **Conclusions:** The growing evidence on elastography has provided standardized reference values, further enhancing its clinical applicability in NICU settings.

## 1. Introduction

Ultrasound is an important imaging technique for diagnosing liver pathologies in neonates and pediatric patients and monitoring and assessing responses to treatment in some of these conditions. It allows the evaluation of ultrasound morphology, structure, and liver vascularization and detects malformation abnormalities or the presence of nodules or masses [1]. However, conventional ultrasound has limitations in diagnosing diffuse liver disease because these conditions involve changes in the echostructure, which can show slight variations depending on physiological conditions, the ultrasound machine, the probe, and the program used. Usually, these subtle variations do not indicate pathology. Therefore, their assessment is highly subjective and relies not only on these factors but especially on the operator’s experience in detecting disease [1].

On the other hand, one of the classic methods of detecting liver pathology is palpation of the organ. This method can assess size increases, masses, and standard stiffness or hardness. However, this assessment also depends heavily on the neonatologist’s experience and only allows a superficial evaluation of the organ [2].

Elastography addresses these limitations by assessing tissue stiffness, not just on the organ’s surface, which changes due to various physiological and pathological mechanisms [2,3]. Sonoelastography involves integration with B-mode ultrasound, so guided by the anatomical ultrasound image, it is possible to target the area where elastography measurements should be taken precisely, thus avoiding vascular structures or, on the other hand, targeting selected regions that are suspected to be more affected in cases of heterogeneous liver involvement [2,3].

Elastography techniques are integrated into the image, with the most widely used currently being the one that generates pulses of acoustic radiation force (ARFI) within the probe itself. This includes the measurement of shear wave velocity at a specific point in the organ, referred to as point shear-wave elastography (pSWE), and assessment in an area known as two-dimensional shear-wave elastography (2D-SWE) [4,5,6,7]. Today, several manufacturers implement ARFI technology (both pSWE and 2D SWE) in their ultrasound devices and offer recommendations for technique optimization and data quality assessment [7,8].

The use of liver elastography in neonates admitted to intensive care units is increasing. This technique has been used to evaluate liver diseases derived from hypoxic-ischemic conditions, cholestatic disorders such as biliary atresia, and diffuse liver involvement caused by infectious, metabolic, or storage disorders [1,9].

However, no publication has described the specific considerations when conducting this point-of-care study for neonatal or intermediate intensive care units. This is especially relevant because the information obtained through ultrasound and its complementary tools can enhance the initial management and treatment monitoring of various pathologies.

In addition, biopsy is the gold standard for detecting diffuse liver pathology. Although elastography does not replace this method, conventional ultrasound and elastography can help decide when to biopsy and direct the biopsy to the most pathologic areas in cases of heterogeneous liver involvement [2].

This study aims to describe the point-of-care evaluation of liver ARFI sonoelastography in neonates within intensive and intermediate care units.

## 2. Technique

Typically, patients in neonatal intensive and intermediate care units can undergo conventional B-mode liver ultrasound and pSWE elastography using a 1–6 MHz convex probe, along with 2D-SWE elastography employing both 1–6 MHz convex and 12–14 MHz linear probes. In our case, we carried out this procedure with a Samsung RS85 Prestige^®^ ultrasound scanner (Samsung Medison Co., Ltd., Seoul, Republic of Korea ).

**Figure 1:**
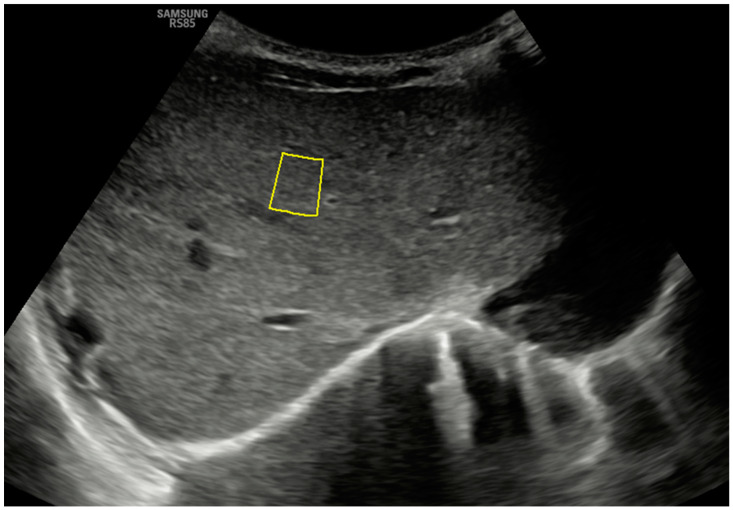
A B-mode ultrasound image in the hepatic anatomical plane focuses on segment IV and shows the correct positioning of the ROI for pSWE with a convex probe. Note the quality measurement (on this device, RMI) of 0.7.

To perform these elastography procedures, both probes are placed transversely in a subcostal location along the right parasagittal midline, where segment IV of the liver can be visualized. Figure 1 shows the B-mode anatomical plane obtained using this approach, corresponding to an oblique axial plane just below the hepatic veins, and how the pSWE measurement is taken. The region of interest (ROI) should be positioned in the center of the ultrasound beam, within 1 cm of the liver capsule and up to 8 cm from the skin’s surface. ROIs are placed at different depths, and the measurement must have an internal quality factor (Reliability Measurement Index or RMI on our ultrasound machine) greater than 0.4 (range 0.0 to 1.0) to be considered adequate. It is desirable to obtain at least 10–15 adequate measurements so that the program will calculate the average obtained in kilopascals (kPa) and meters per second (m/s). The obtained average will only be considered representative if the interquartile range of the suitable measures obtained, divided by the median of this average, is less than 30% for the kPa parameter and below 20% for the m/s parameter (ideally below 20% and 10%, respectively) [2,3].

**Figure 2:**
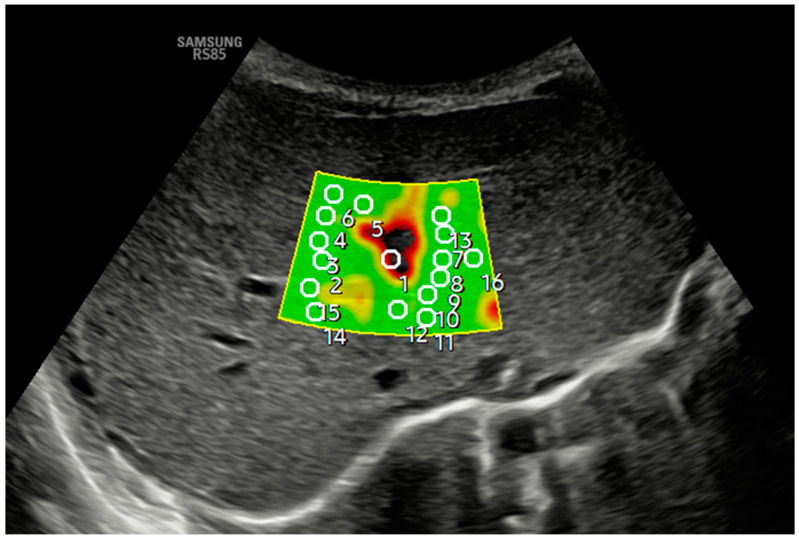
An axial section in segment IV obtained a B-mode ultrasound image in the liver anatomical plane, and samples were acquired with 2D-SWE using a convex probe. The measurement area is positioned, and the shear wavefront color map is generated. The color map, or elastogram, can be adjusted according to the color code assigned by each commercial house from highest to lowest stiffness or, as in the example, according to the quality of the measurement (in the example, green is optimal or very optimal measure quality), always selecting the one with the fewest artifacts and using the lowest possible ROI size. At least 10–15 valid measurements are obtained within the elastogram using the same validity criteria mentioned in the main text for pSWE. In the example, ROI 1 is invalid.

**Figure 3:**
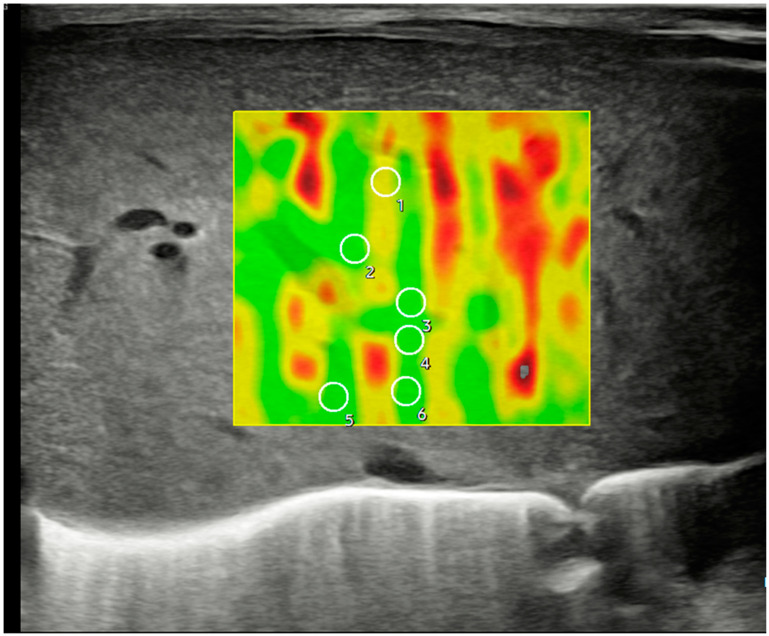
Ultrasonographic example of a hepatic axial section in segment IV with sample acquisition using 2D-SWE and a linear probe, following the same methodology described in Figure 2.

Figure 2 and Figure 3 show how 2D-SWE is performed with convex and linear probes. The measurement area is positioned with probes, generating a color map of the shear wavefront. The color map, or elastogram, can be adjusted as needed, ideally always selecting the one with the fewest artifacts. We use the smallest possible ROI and obtain at least 10–15 valid measurements within the elastogram, applying the same validity criteria as pSWE.

## 3. Peculiarities in ICUs and Neonatal Care Units and Discussion

There are two critical factors when performing sonoelastography in neonates: weight and gestational age, which reflect the maturity of patients and their tissues. The operator should consider these factors as they substantially influence the ultrasound images to achieve optimal sonoelastography. Neonates weighing less than 1500 g at birth, especially those weighing less than 1000 g, are extremely sensitive to touch, external noise, and temperature changes and susceptible to infections. Consequently, strict hygiene and aseptic measures must be implemented during the examination and handling of the tubes. In general, and in the experience of the authors, the following points are recommended:

1. It is crucial to minimize patient contact time to maintain their overall stability and an aseptic environment. Elastography should take no longer than 1 to 2 min, with the mechanical index (MI) and thermal index (TI) values set to the lowest possible settings from the beginning (ideally below 0.4 MI) for biosecurity reasons [10], ensuring that the total scan time, including elastography, does not exceed 5 to 6 min.
2. The patient must remain as comfortable as possible during the exam. Nurses play a vital role in improving patient comfort and ensuring the proper functioning of any respiratory, digestive, and vascular devices the patient may have. This is especially important in intensive care units, where at least one staff member must always be at the patient’s bedside.
3. The patient should be examined supine, ideally without removing the kangaroo from its crib or incubator. The patient should remain calm and stable, avoiding interruptions of kangaroo care or feeding hours.
4. Unlike older children or adolescents, neonates are not allowed to fast for extended periods, as irritability and crying would make the examination impossible. Therefore, elastography should be performed at the most convenient time, regardless of the fasting state. The patient should be in a postprandial state to ensure peace of mind, thus minimizing the time of exploration. There is ample literature on other age groups that shows fasting does not significantly affect the stiffness values obtained through elastography [11,12].
5. In more minor patients (<1000 g), obtaining sufficient measurements is often more difficult, as the available depth cannot exceed 3 cm. To address this, we recommend using contact surface expansion devices, such as ultrasound gel pads, to increase the depth or, more simply, take measurements of the left hepatic lobe. This measurement has not significantly altered the values obtained in other age groups [11,12].
6. Elastography can be challenging in patients undergoing high-frequency oscillatory ventilation (HFO), a treatment for respiratory failure in preterm infants. In some cases, the exam length can increase significantly, from less than a minute to 14 minutes. In other situations, obtaining a valid measurement may not be possible at all. Therefore, especially if the patient is hemodynamically unstable, the examination should only be performed if it is essential for their management.

## 4. Conclusions

Based on our experience performing ultrasound scans on patients in neonatal intensive care units, we have found sonoelastography to be easy to use, reproducible, portable, and highly beneficial in routine practice when assessing liver pathology. Its advantage lies in the absence of ionizing radiation. When performing sonoelastography in these patients, it is essential to consider the specific factors described in this article and refer to the normalized values published in the relevant literature.

## Data Availability

Data used to support the present study’s findings are available upon request from the relevant author.
